# Weigh-in-Motion: Lightweight Real-Time Identification of Gbps Wireless Traffic

**DOI:** 10.3390/s22020437

**Published:** 2022-01-07

**Authors:** Sungsoo Kim, Joon Yoo, Jaehyuk Choi

**Affiliations:** School of Computing, Gachon University, 1342, Seongnam-daero, Sujeong-gu, Seongnam-si 13120, Korea; xiroxiro@gc.gachon.ac.kr

**Keywords:** packet classification, network monitoring, passive online detection, hypothesis test, 802.11 frame aggregation

## Abstract

Distinguishing between wireless and wired traffic in a network middlebox is an essential ingredient for numerous applications including security monitoring and quality-of-service (QoS) provisioning. The majority of existing approaches have exploited the greater delay statistics, such as round-trip-time and inter-packet arrival time, observed in wireless traffic to infer whether the traffic is originated from Ethernet (i.e., wired) or Wi-Fi (i.e., wireless) based on the assumption that the capacity of the wireless link is much slower than that of the wired link. However, this underlying assumption is no longer valid due to increases in wireless data rates over Gbps enabled by recent Wi-Fi technologies such as 802.11ac/ax. In this paper, we revisit the problem of identifying Wi-Fi traffic in network middleboxes as the wireless link capacity approaches the capacity of the wired. We present Weigh-in-Motion, a lightweight online detection scheme, that analyzes the traffic patterns observed at the middleboxes and infers whether the traffic is originated from high-speed Wi-Fi devices. To this end, we introduce the concept of ACKBunch that captures the unique characteristics of high-speed Wi-Fi, which is further utilized to distinguish whether the observed traffic is originated from a wired or wireless device. The effectiveness of the proposed scheme is evaluated via extensive real experiments, demonstrating its capability of accurately identifying wireless traffic from/to Gigabit 802.11 devices.

## 1. Introduction

The trend of using Bring Your Own Device (BYOD) policies has been increasing over the years, which allows company employees to bring unmanaged personal devices into their workspace and connect to internal networks [1,2]. The recent COVID-19 pandemic and the resulting surge of employees working from home have further increased the need for the BYOD policy. According to Bitglass’s 2021 security report [3], as of 2021, 82% of organizations actively adopt a BYOD-friendly policy. The BYOD offers many benefits including increased employee productivity and greater employee satisfaction, but it can significantly increase security and privacy risks introduced by the use of personal mobile devices within the organization.

One of the most relevant wireless security threats is the rogue access point (rogue AP). A rogue AP is an unauthorized AP connected to an organization’s network, not under the management of the network administrator, often deployed by employees wanting unfettered wireless access [4]. It can also be created by malicious insiders to conduct malicious attacks such as DoS (Denial-of-Service), and data theft, thereby creating a security hazard [4,5]. For this reason, it is critical for most organizations to detect the rogue AP to defend against the ever-increasing potential security threats.

One effective approach to cope with the rogue AP problem is to detect unauthorized wireless usage by identifying wireless traffic at middleboxes in the network. Several existing solutions exploit differences in network statistics, e.g., round-trip-time (RTT) and inter-packet arrival time (IAT), observed in wireless traffic to distinguish ‘slow’ wireless traffic (inferring undiscovered wireless devices, e.g., a rogue AP) from ‘faster’ wired traffic [6,7]. The effectiveness of such approaches relies heavily on their underlying assumption that the wireless link capacity will never reach the capacity of the wired.

However, this assumption is no longer valid due to the technical improvements enabled by recent Wi-Fi technologies. For instance, 802.11ac and 802.11ax provide Gigabit bandwidth performance, up to 1.69 Gbps and 4.8 Gbps, respectively, which exceeds the bandwidth of 1-Gbps Ethernet wired links [8,9].

In this paper, we study the problem of identifying Wi-Fi traffic in network middleboxes as the wireless link capacity approaches the capacity of the wired. We propose a lightweight, online passive-classification method, called Weigh-in-Motion, that can identify Gigabit Wi-Fi traffic in network middleboxes. Weigh-in-Motion introduces a new packet-level feature called ACKBunch that captures the unique characteristics of high-speed Wi-Fi.

We demonstrate the accuracy of our approach by implementing the prototype of Weigh-in-Motion. Our approach is shown to classify Gbps wireless traffic very quickly (the mean of 0.72 s) with high accuracy (more than 99 percent).

We summarize our contributions as follows:

(1) Introduction of a new packet-level feature easily measurable at middleboxes for run-time packet classification of Gigabit speed wireless traffics (see Section 4).
(2) Development of a lightweight run-time traffic classification algorithm using the sequential hypothesis testing (see Section 5).
(3) Verification of the proposed algorithm through extensive real-world experiments and prototype implementation (see Section 6).

The rest of the paper is organized as follows. We discuss the related work and illustrate the preliminaries in Section 2. We formulate the problem in Section 3. We present a new concept of ACKBunch in Section 4. Section 5 presents the proposed framework. We demonstrate the evaluation result in Section 6 and discuss our approach in Section 7. Finally, Section 8 concludes this work.

## 2. Background

In this section, we overview related work on traffic classification methods and then introduce the background of high-speed 802.11 networks.

### 2.1. Related Work

A variety of methods [6,7,10] have been proposed to address the problem of identifying Wi-Fi traffic based on passive and run-time measurements. Most existing approaches employ statistical quantities (e.g., the magnitude of deviation, mean, median, and so on) of the delay-based network metrics such as inter-packet arrival time (IAT) and round-trip-time (RTT). In Section 3.2, we present the limitations of these approaches for packet classification in high-speed wireless access networks. On the other hand, our approach can correctly conduct packet classification using ACKBunch as described in Section 4.

Research on traffic classification using Machine Learning (ML) has obtained growing attention. The work in [11] uses Software Defined Networking (SDN) and OpenFlow (OF) protocol to classify enterprise network traffic. They leverage Machine Learning to extract knowledge from the collected data. The results show high accuracy for some applications (e.g., Web traffic), but poor performance for other ones (e.g., LinkedIn). In [12], supervised Support Vector Machine (SVM) and unsupervised K-means clustering are studied for traffic classification in SDN networks. They show that machine learning can achieve good accuracy. In [13], the authors have presented a system for classifying user activities from network traffic using both supervised and unsupervised learning. The proposed system exploits the behaviour exhibited over the network and classifies the underlying user activity, taking into consideration all of the traffic generated by the user within a given time window. The approach proposed in [14] uses fingerprints for authentication and identification purposes by training an ML method based on multiple network traffic features to distinguish similar device types. The authors [15] have proposed deep-learning based network Traffic classifiers with Convolutional and Recurrent Neural Networks (CNN and RNN) for Internet of Things.

### 2.2. Frame Aggregation in High-Speed IEEE 802.11 Standards

IEEE 802.11ac and 802.11ax [8,9], also known as Wi-Fi 5 and 6, respectively, are the de-facto Wi-Fi networking standards, which promise gigabit wireless link speeds and many enhancements over the previous 802.11n technology. We first discuss some of the key features supported by 802.11ac/ax for improving performance over earlier standards.

The key feature of the 802.11ac/ax exists at the physical (PHY) layer, which employs advanced signal processing and modulation techniques and multiple antennas and wider channels, offering a significant increase in the maximum PHY data rate up to 6.933 Gbps and 9.6 Gbps, respectively, with the use of multiple spatial streams (e.g., 8 streams) and wider (e.g., doubled) channel bandwidths [8,9,16]. 802.11ac operates only in the less crowded 5 GHz and IEEE 802.11ax is designed to operate in both 2.4 GHz and 5 GHz spectrum bands.

**Figure 1:**
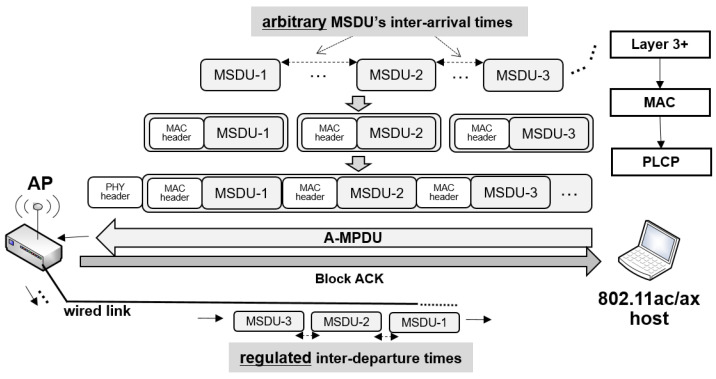
IEEE 802.11 aggregate MAC protocol data unit (A-MPDU) scheme.

Another key MAC-layer enhancement improving network efficiency in 802.11ac/ax is the aggregate medium access control (MAC) protocol data unit (A-MPDU) scheme. In 802.11ac/ax, all frames transmitted use the A-MPDU format. Although 802.11ac does not add any new aggregation methods compared to 802.11n, 802.11ac adds a new take on aggregation: all frames transmitted use the A-MPDU format. Thus, even a single MPDU frame is transmitted as an aggregate frame in 802.11ac [8]. With A-MPDU, multiple MAC-level protocol data units (MPDUs) or subframes are combined into a single PHY-layer protocol data unit (PPDU) for transmission, as shown in Figure 1. If A-MPDU transmission is successfully received, the receiver replies with a Block ACK that contains a bitmap field to indicate the correctly received sub-frames in the A-MPDU frame, and thus each subframe can be retransmitted individually. A-MPDU significantly improves MAC efficiency and reduces protocol overhead such as access delay and several inter frame spaces (IFSs) [16].

802.11ax introduces two major enhancements at the Physical layer [17]; (i) Orthogonal Frequency Division Multiple Access (OFDMA) that can be used in both downlink (DL) and uplink (UL) directions, and (ii) UL MU-MIMO which allows multiple stations to transmit simultaneously over the same frequency resource to the AP. However, there is a lesser enhancement in A-MPDU. The main difference between IEEE 802.11ac and IEEE 802.11ax is that IEEE 802.11ax allows up to 256 MPDUs in an A-MPDU frame whereas IEEE 802.11ac only allows up to 64 MPDUs. For this reason, most experiments in this work have been conducted with off-the-shelf 802.11ac devices.

## 3. Problem Description

We first provide the system model, give the problem formulation considered in the paper, and then explain the limitation of previous approaches.

### 3.1. Problem Statement

**Figure 2:**
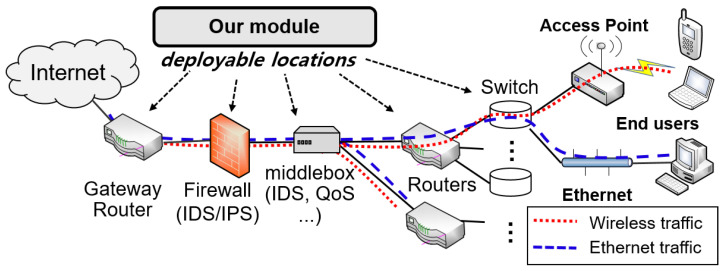
System Architecture.

We consider a general managed network architecture such as enterprise and campus networks, as depicted in Figure 2, where the network is operated, maintained and monitored by network administrators. We assume that end users in the network use either (i) wired Ethernet or (ii) 802.11 Wi-Fi APs to access the Internet. The mixture of wired and wireless traffics coming in (inbound) and going out (outbound) of the network are aggregated and go through the limited number of central gateway or core routers. As in [18,19] and used in the typical Intrusion Detection System (IDS) architecture [20], we assume that our monitoring module passively captures in- and outbound passing traffic either on top of each router or network middleboxes such as intrusion detection systems and application-level firewalls [21].

In this work, we focus on the following problem: “Given observations of a mixture of wired and wireless traffic, can we identify the traffic transmitted over Gigabit Wi-Fi network?” To answer this question, we aim to design an online lightweight traffic classifier that analyzes the traffic patterns observed at the monitoring modules and identifies whether the traffic originated from a Gbps Wi-Fi network such as 802.11ac or from a wired network such as Ethernet. The identified Wi-Fi network can be further tested by using certain existing security solutions such as intrusion detection systems and application-level firewalls (e.g., [20,21,22]) automatically or manually, and classified as a rogue AP or a legitimate AP, where this final step is out of the scope of this paper as it is readily available with existing solutions.

### 3.2. Limitation of Previous Approaches

To motivate the need for our solution, this section highlights the limitation of previous approaches [6,7,10]. In particular, we study the effectiveness of existing schemes via experiments for high-speed wireless access networks such as Wi-Fi 6 whose capacity reaches the capacity of wired link.

We conduct measurements with three network statistics, namely, inter-packet arrival time (IAT), round-trip-time (RTT) and the interarrival time of two consecutive TCP ACK packets (Inter-ACK time) [6], for several types of access networks. Specifically, we look at 100 Mbps Ethernet, 1Gps Ethernet, 802.11g, 802.11n, and 802.11ac networks.

**Figure 3:**
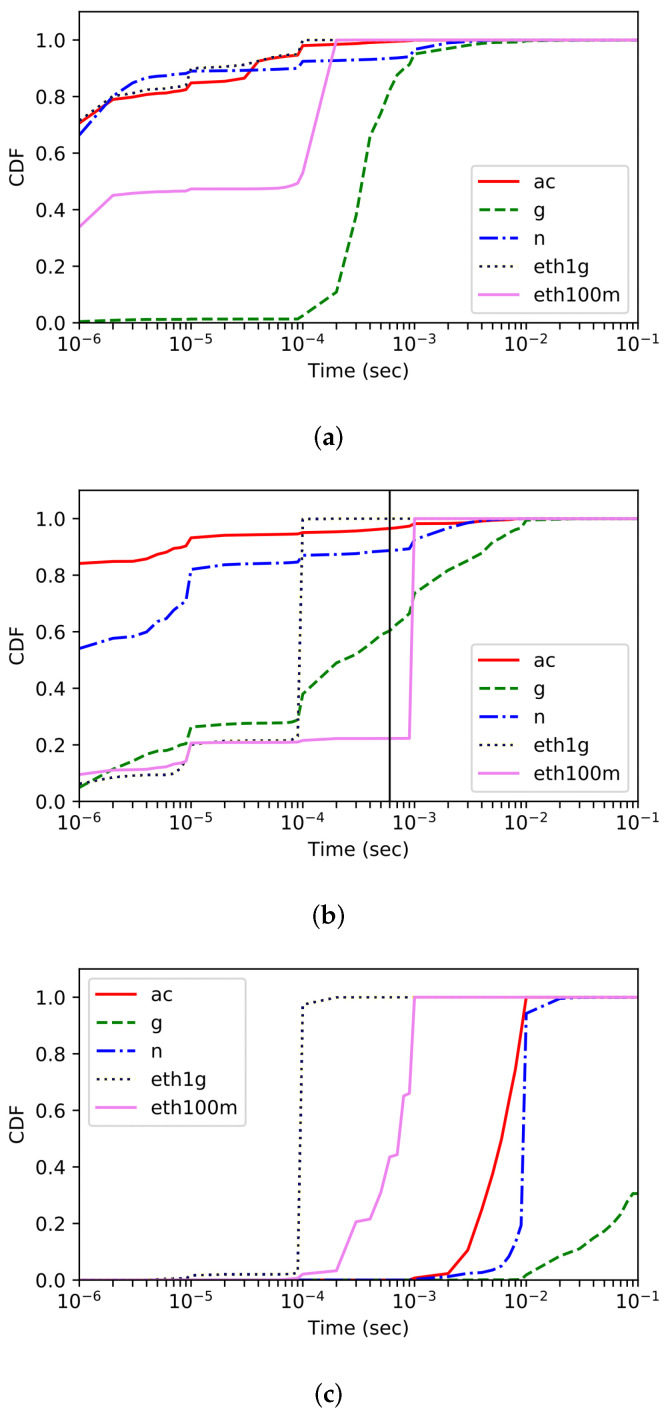
CDF of (**a**) Inter-packet arrival time (IAT), (**b**) Inter-ACK time, and (**c**) round-trip-time (RTT).

Figure 3a–c show the cumulative distribution functions (CDFs) of the IAT, RTT, and Inter-ACK time, respectively. Figure 3a shows that it is difficult to distinguish IAT’s distribution between high-speed Wi-Fi and Ethernet, explaining why the delay-based approach does not work properly for today’s Gbps Wi-Fi traffic while an old Wi-Fi technology, i.e., 802.11g, traffic is easy to distinguish. Similarly, as shown in Figure 3b, the inter-ACK time corresponding to the 802.11ac traffic is difficult to distinguish itself from that of the Ethernet traffic. As a result, while the scheme proposed in [6] is effective in distinguishing the traffic arriving from older Wi-Fis, such as 802.11a, 802.11b, and 802.11g, it is likely to classify the 802.11ac traffic as Ethernet one.

Figure 3c illustrates that the probability distribution of RTT shows better performance in differentiating between Wi-Fi and Ethernet, implying that it can be used as a metric for classification. However, the RTT-based approach is stateful, which should remember the TCP packet (i.e., unacked data) to match TCP-ACK pairs. It may require large memory space and high computation costs, which becomes a non-negligible burden on the monitoring module in practice. Besides, as pointed in [10], it is known that RTT is sensitive to environmental variables such as background traffic and congestion levels of networks, making it difficult to guarantee high classification accuracy. In addition, as wireless technologies advance, the delay on the wireless link is gradually decreasing, and the RTTs of Wi-Fi traffic are getting closer to the RTTs of wired traffic. Due to these limitations of using RTT for traffic classification, we incorporate a new lightweight and accurate approach in our work.

## 4. A New Lightweight Feature

In this section, we introduce a new simple and practical metric, namely, called *ACKBunch*, that can characterize wireless traffic from high-speed 802.11 devices. We then describe the characteristics and the main observations regarding *ACKBunch* through extensive experiments with real measurements, which will be exploited to differentiate high-speed wireless traffic from wired Ethernet traffic in an online manner.

### 4.1. Observation

Our key observation is that frame aggregation, an essential mechanism of high-speed Wi-Fi standards including IEEE 802.11n, 802.11ac, and 802.11ax, imposes *traffic shaping effect* or packet bunching effect. Note that in network systems, traffic shaping is a technique that delays the flow of certain types of packets to bring them into compliance with the desired traffic profile [23]. That is, although traffic shaping is not explicitly used in 802.11, multiple outbound wireless packets are *shaped* into a single large size Bunch, i.e., A-MPDU frame, to be transmitted. Then, the packets received at the AP are individually forwarded to the next wired-side hop over a very short time period with very short inter-packet departure intervals, as illustrated in Figure 1.

**Figure 4:**
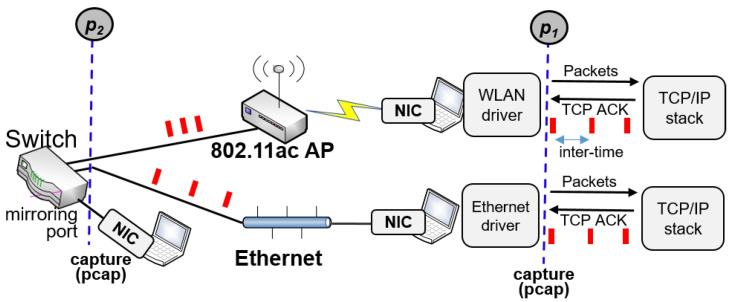
Experimental setup.

**Experiment:** To understand and verify the packet bunching effect (or traffic shaping) due to frame aggregation mechanism, we conducted measurement studies as shown in Figure 4.

Figure 4 describes the experimental environment. For Ethernet and 802.11ac network interface cards (NICs), we have captured all outgoing packets from each of them using Wireshark [24] at two different monitoring points ($p_{1}$ and $p_{2}$ in Figure 4), respectively, under saturated traffic conditions. As described in Section 2.2, IEEE 802.11ax and 802.11ac use the same A-MPDU scheme except for the maximum number of MPDUs allowed in a A-MPDU frame. For this reason, we focus on the IEEE 802.11ac and have conducted most experiments with off-the-shelf IEEE 802.11ac devices. Note that for the experiment we used the same laptop (i7-CPU, 8 GB memory) equipped with both a 10 Gbps Ethernet and an 802.11ac NIC, so all the other environmental variables in each experiment are the same except for the NIC. We measured the time difference or inter-arrival time $\Delta t_{p}^{\left(i\right)}=t_{p}^{\left(i\right)}-t_{p}^{\left(i-1\right)}$, where $t_{p}^{\left(i\right)}$ denotes the arrival time of *i*-th ACK of a target TCP flow at two monitoring points $p\in \{p_{1},p_{2}\}$ in Figure 4.

We then observed the change in the inter-arrival times for two consecutive outgoing packets moving from $p_{1}$ to $p_{2}$ by calculating the difference $\Delta t_{p1}^{\left(i\right)}-\Delta t_{p2}^{\left(i\right)}$.

**Figure 5:**
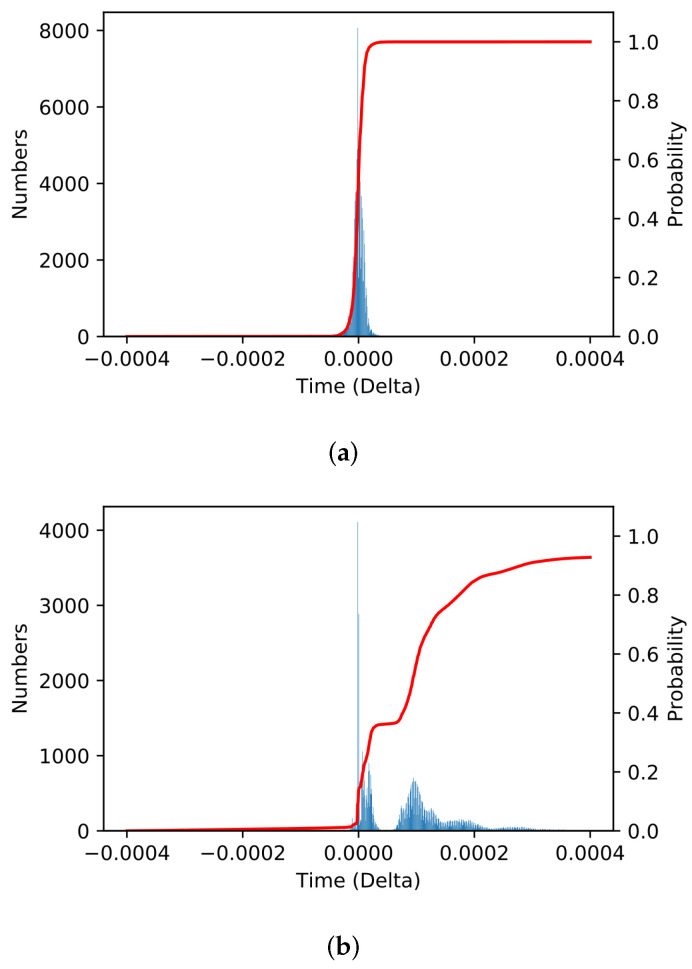
Distribution (pdf and cdf) of time difference of inter-packet times $\Delta t_{p1}-\Delta t_{p2}$ for (**a**) Ethernet and (**b**) 802.11ac traffics.

**Results:**Figure 5a,b show the probability functions (pdf and cdf) of the time difference $\Delta t_{p1}^{\left(i\right)}-\Delta t_{p2}^{\left(i\right)}$ for Ethernet and 802.11 ac traffics, respectively. Figure 5a shows a normal distribution with zero mean, implying that there are no explicit changes (except minor random fluctuations) in the inter-packet times of Ethernet traffic.

On the other hand, the experimental results for 802.11ac traffics show that the inter-packet time was significantly reduced at p2 compared to ones measured before wireless transmission at p1 as shown in Figure 5b. It implies that *packets transmitted over the 802.11ac link are aggregated by A-MPDU frame aggregation mechanism, forming a unique inter-packet time distribution or traffic profile different from those of the wired traffic*. Recall that all 802.11ac frames are transmitted using the A-MPDU format.

### 4.2. ACKBunch: A New Lightweight Network Statistic

Motivated by the observation in the previous subsection, we first define the ACKBunch as follows.

**Definition** **1.** *(ACKBunch). A bunch or set of TCP ACK packets observed within a certain time range at a monitoring point is said to be an ACKBunch, where all the TCP ACK packets belong to the same TCP flow and are arrived within a certain time range. Let $\Lambda^{m}$ be an ACKBunch for TCP flow m. An ACKBunch can be represented with the ordered n-tuple $\Lambda^{\left(m\right)}=\left(a_{1}^{m},a_{2}^{m},\ldots ,a_{n}^{m}\right)$ where $a_{i}$ represents n-th element, i.e., TCP ACK packet*.

*The inter-packet arrival time between any two consecutive packets in an ACKBunch should be smaller than a certain value, called partition threshold δ. That is, a set of TCP ACK packets $\left(a_{1}^{m},a_{2}^{m},\ldots ,a_{n}^{m}\right)$ forms a ACKBunch $\Lambda^{\left(m\right)}$ if and only if $t\left(a_{i+1}^{m}\right)-t\left(a_{i}^{m}\right)<\delta$ for ∀i∈{1,2,3,…,n−1}, where m denotes the TCP flow index and $t(a_{i}^{m})$ is the arrival time of i-th ACK of TCP flow m*.

**Definition** **2.** *(ACKBunch Size or Weight). For $\Lambda^{\left(m\right)}=\left(a_{1}^{m},a_{2}^{m},\ldots ,a_{n}^{m}\right)$, we say that n is the size or length of ACKBunch $\Lambda^{\left(m\right)}$ which represents the number of TCP ACK packets included in the ACKBunch. The size of ACKBunch is denoted by $|\Lambda^{\left(m\right)}|$*.

**Figure 6:**
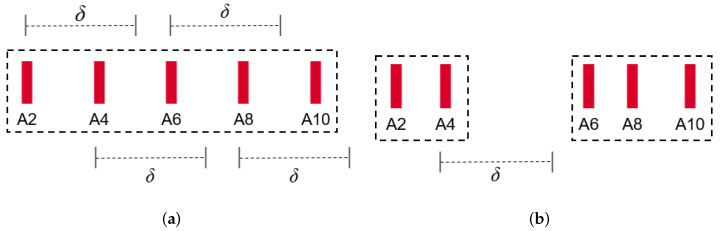
Examples of ACKBunch. (**a**) one ACKBunch with 5 ACKs, (**b**) two ACKBunches each with 3 and 2 ACKs, respectively.

We give a few examples of ACKBunch. In Figure 6a, the four ACKs A4, A6, A8, and A10 arrive within δ of the previous ACK’s arrival, thus forming an ACKBunch of size 5. In Figure 6b, A4 and A6 arrive within δ of the previous ACK’s (A2 and A4, respectively) arrival, but the fourth ACK (A8) arrives after δ of the previous A6. The next ACK (A10) arrives within δ of the previous A8. Therefore, two ACKBunches are formed each with size 3 and size 2.

In order for two different transmissions to be considered as separate ACKBunches, the *partition threshold* value must be defined properly. Based on the 802.11 standard, we derive the following proposition which can act as an minimum guideline to determine the partition threshold.

Clearly, the *partition threshold* δ is the most critical factor that determines the ACKBunch size. A large δ value will give a larger ACKBunch size, and vice versa. In order to determine the appropriate partition threshold δ value, we utilize the Inter-Frame Space (IFS) time in the IEEE 802.11 Distributed Coordination Function (DCF).

We give a brief description of DCF as follows. In DCF, each station will check if the channel is busy, in which case it will wait, then if the channel becomes idle, then it will defer access for at least a IFS time duration. The IFS time will defer depending on what type of frame the station is transmitting. SIFS (Short IFS) is the shortest time, thus highest priority, and is used by Clear-to-Send (CTS) and ACK frames. DIFS (DCF IFS) is used for general Request-to-Send (RTS) or data frames.

In this paper, we set the partition threshold δ to the DIFS value. The reasons are twofold. First, it can prevent the ACK-Bunch from growing too large. Second, by setting δ to the DIFS value, the TCP ACK packets that are included as subframes in an A-MPDU frame will be incorporated in a single ACKBunch. This will clearly help differentiating between Ethernet and IEEE 802.11ac packets.

Table 1 shows the DCF slot time and DIFS values categorized by different IEEE 802.11 standards. We observe that the DCF slot time and DIFS values differ for each standard. For example, the DIFS value of IEEE 802.11n is 28 or 50 μs for the 2.4 GHz frequency, but 34 μs for the 5GHz frequency. In our work, we set the partition threshold δ to the median value of 34 μs.

### 4.3. Distribution of ACKBunch Size

**Figure 7:**
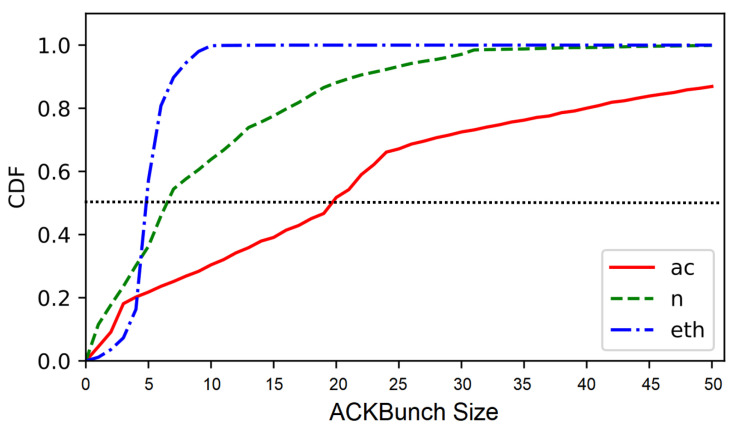
Distribution of the *ACKBunch* sizes for wired traffic (Ethernet), legacy Wi-Fi traffic (802.11n) and high-speed Wi-Fi traffic (802.11ac).

Figure 7 shows the c.d.f. of the size of the proposed ACKBunch for wired traffic (Ethernet), legacy Wi-Fi traffic (802.11n) and high-speed Wi-Fi traffic (802.11ac). As discussed in Section 3.2, the delay-based network metrics such as inter-packet arrival time (IAT) and round-trip-time (RTT) have limitations to distinguish Gbps wireless traffic from Ethernet traffic. Meanwhile, as expected, ACKBunch is shown to have higher probabilities for larger sizes than the wired traffic and legacy Wi-Fi traffic, indicating that its unique pattern can be exploited to classify Gbps Wi-Fi traffics.

In what follows, we will detail how ACKBunch is a statistic that can be used as a new metric that can be used to effectively classify high-speed wireless traffic from wired traffic.

## 5. Weigh-in-Motion: Online Detection Strategy

We now present a simple online algorithm that passively monitors outbound TCP-ACK streams and identifies Gbps Wi-Fi traffic in network middleboxes.

### 5.1. Criterion for Identifying Gbps Wi-Fi Traffic

Our key observation to identify Gbps Wi-Fi traffic is to exploit the empirical distribution of *ACKBunch* size |Λ| shown in Figure 7. As discussed above, for a given TCP flow *m* from 802.11ac, we have a higher empirical probability of $Pr(|\Lambda^{\left(m\right)}|>k)$, the probability that *ACKBunch* size is larger than *k*, than those of Ethernet traffic and typical Wi-Fi traffic. For instance, $Pr(|\Lambda^{\left(m\right)}|>10)$, the probability that *ACKBunch* size is greater than *k* = 10 is close to zero in Ethernet traffic, while 802.11ac traffic has a relatively large value as shown in Figure 7. Also, the median value of *ACKBunch* size for Gbps Wi-Fi traffic is about 19, while the median for Ethernet traffic is about 5.

Based on this observation, we cast the classification problem as a hypothesis testing with two hypotheses, $H_{0}$ (null hypothesis) and $H_{a}$ (alternative hypothesis), where $H_{a}$ represents that the observed traffic is Gbps Wi-Fi traffic. Given target TCP flow *m*, we state the hypothesis testing problem as

(1) $$\left\{\begin{array}{c} H_{0}:Pr(|\Lambda^{\left(m\right)}\left|>k\right)\leq \theta \;\left(\mathrm{not}\mathrm{Gbps}\mathrm{Wi}\text{-}\mathrm{Fi}\mathrm{traffic}\right),\\ H_{a}:Pr(|\Lambda^{\left(m\right)}\left|>k\right)>\theta \;\left(\mathrm{Gbps}\mathrm{Wi}\text{-}\mathrm{Fi}\mathrm{traffic}\right), \end{array}\right.$$

or

(2) $$\left\{\begin{array}{c} H_{0}:Pr(|\Lambda^{\left(m\right)}\left|\leq k\right)>\theta_{0}:=1-\theta \;\left(\mathrm{not}\mathrm{Gbps}\mathrm{Wi}\text{-}\mathrm{Fi}\mathrm{traffic}\right),\\ H_{a}:Pr(|\Lambda^{\left(m\right)}\left|\geq k\right)\leq \theta_{0}:=1-\theta \;\left(\mathrm{Gbps}\mathrm{Wi}\text{-}\mathrm{Fi}\mathrm{traffic}\right). \end{array}\right.$$

Here, we denote $\theta_{0}$ as the probability that *ACKBunch* size is less than or equal to *k*. For evaluation in Section 6, we will use θ = $\theta_{0}$ = 0.5 for *k* = 12 based on the empirical distribution of *ACKBunch* size |Λ| shown in Figure 7.

### 5.2. Online Algorithm with Sequential Hypothesis Test

We present a lightweight online algorithm that identifies Gbps Wi-Fi traffic using a sequential hypothesis testing [25], based on the decision criterion in Equation (2).

Suppose we have *n* observed sequence of $\Lambda^{\left(m\right)}$ of target TCP ACK flow *m*, denoted by $\Delta^{m}=\left\{\right|\Lambda_{1}^{m}\left|,\ldots ,\right|\Lambda_{n}^{m})|\}$ at a middlebox. Let $\theta_{0}=Pr(|\Lambda^{\left(m\right)}\left|\leq k\right)$ denote the probability that *ACKBunch* size is less than or equal to *k*.

If TCP-ACK flow *m* is not a high-speed Wi-Fi traffic (e.g., Ethernet or legacy Wi-Fi), its observed sequence $\Delta^{m}=\left\{\right|\Lambda_{1}^{m}\left|,\ldots ,\right|\Lambda_{n}^{m})|\}$ will satisfy the hypothesis $H_{0}:Pr(|\Lambda^{\left(m\right)}\left|\leq k\right)\leq \theta_{0}$ in Equation (2). Hence, if the null hypothesis $H_{0}$ is *rejected* by the observed sequence $\Delta^{m}=\left\{\right|\Lambda_{1}^{m}\left|,\ldots ,\right|\Lambda_{n}^{m})|\}$, we can conclude that flow *m* is a Gbps Wi-Fi traffic.

To design an online decision algorithm based on passive observation to identify Gbps Wi-Fi traffic, we adopt a sequential hypothesis test method, in particular, the *likelihood ratio test* (LRT) [26] and assess the goodness of fit of two statistics, i.e., hypothesis and observation.

Let $X_{1},X_{2},\ldots ,X_{n}$ be random samples from independent and identically distributed(i.i.d.) discrete observations for TCP-ACK flow *m*. For the given observed $\Delta^{m}=\{X_{1}=\left|\Lambda_{1}^{m}\right|,\ldots ,X_{n}$$=|\Lambda_{n}^{m})|\}$ with a parameter $\theta_{0}$ its likelihood ratio is defined as

(3) $$L\left(\theta_{0}|\Delta^{m}\right)=\prod_{i=1}^{n}f(|\Lambda_{i}^{m}\left|;\theta_{0}\right).$$

One way to test $H_{0}$ vs. $H_{a}$ is to compare the corresponding likelihood functions. If we assume $H_{0}$ were correct, the likelihood ratio test (LRT) statistic is given as

(4) $$LR=\frac{\sup_{H_{0}}L\left(p|\Delta^{m}\right)}{\sup_{H_{a}}L\left(p|\Delta^{m}\right)}=\frac{\sup_{0\leq p\leq \theta_{0}}L\left(p|\Delta^{m}\right)}{\sup_{0\leq p\leq 1}L\left(p|\Delta^{m}\right)},$$

where the numerator $\sup_{H_{0}}L\left(p|\Delta^{m}\right)$ is the maximum likelihood that the observed sequence $\Delta^{m}$ is in $H_{0}$, and $\theta_{0}=Pr(|\Lambda^{\left(m\right)}\left|\leq k\right)$ denotes the probability that *ACKBunch* size is less than or equal to *k*. The denominator $\sup_{H_{a}}L\left(p|\Delta^{m}\right)$ represents the maximum probability of the observed sequence in all possible cases or in the alternative hypothesis $H_{a}$ (i.e., Gbps Wi-Fi traffic).

Note that the maximum likelihood estimator (MLE) in the numerator is $\min\{p,\theta_{0}\}$ while the denominator has *p* as the MLE. Therefore, if LR=1, then we can say that the most likely value of *p* is in the null hypothesis and we should not reject $H_{0}$ in Equation (2). On the other hand, is LR is much smaller than 1, the alternative hypothesis $H_{a}$ is more likely with the observed sequence than the null hypothesis $H_{0}$. Thus, the LRT will reject if:

(5) $$LR<\frac{1}{K},$$

where K∈R is the predefined decision parameter. We will study the impact of *K* on the detection performance on the detection accuracy and detection time in Section 6, where we will use $K=10^{4},10^{5},\mathrm{and}10^{6}$.

***Weigh-In-Motion-*****Online Sequential Test Algorithm:** Based on the LRT, we now formulate the online classification problem for TCP-ACK flow *m* as a sequential hypothesis test. Let $\hat{p}$ denote the MLE of *p*, i.e., the ratio of the *ACKBunch* size less than or equal to *k*, to all observed sequences. In particular, for total *n* observed *ACKBunch* sequences, *l* *ACKBunch* observations are less than or equal to *k*, then $\hat{p}$ is given by $\hat{p}=l/n$. Then, the LRT given in Equation (4) becomes

(6) $$LR=\frac{\sup_{0\leq p\leq \theta_{0}}p^{l}{\left(1-p\right)}^{n-m}}{\sup_{0\leq p\leq 1}p^{l}{\left(1-p\right)}^{n-m}}.$$

For $\theta_{0}<\hat{p}<1$, the LRT rejects $H_{0}$ and concludes that the traffic is Gbps Wi-Fi traffic if    

$$\frac{\theta_{0}^{l}{\left(1-\theta_{0}\right)}^{n-l}}{{\hat{p}}^{l}{\left(1-\hat{p}\right)}^{n-l}}<\frac{1}{K},$$

that is

(7) $$n<\frac{l\left(\log\left(\frac{\hat{p}}{1-\hat{p}}\right)+log\left(\frac{1-\theta_{0}}{\theta_{0}}\right)\right)-\log K}{\log\left(1-\theta_{0}\right)-\log\left(1-\hat{p}\right)}.$$

Based on the rigorous analysis, we will use *k* = 12 and $\theta_{0}=0.5$.

Algorithm 1 describes the procedure for the aforementioned sequential hypothesis testing. Procedure *Initialize()* initializes all the parameters. Procedure *OnReceiveTCPAck()* runs when a new TCP ACK is received: lines 3 to 6 show the case when a TCP-ACK belongs to the ACKBunch, and lines 7 to 24 represent when the ACKBunch is separated, and the procedure *TestSPRT(n, l)* is run. In procedure *TestSPRT(n, l)* lines 1 to 7, Equation (7) is tested to conclude if the flow belongs to the Gbps Wi-Fi traffic or not.

**Enhanced Weigh-In-Motion Algorithm:** As will be shown later in Table 2, we have observed that some inaccuracies existed for Algorithm 1. In order to determine its underlying causes, we conducted a deeper investigation on the effect of the network configuration environment of the 802.11 host. As a result, we found that TCP’s congestion control algorithm greatly affects the accuracy of Algorithm 1. In other words, the distribution of ACKBunch sizes in the *Compound TCP* congestion control algorithm widely used in Microsoft Windows OS [27] shows a different distribution pattern than other TCP congestion control algorithms.

**Figure 8:**
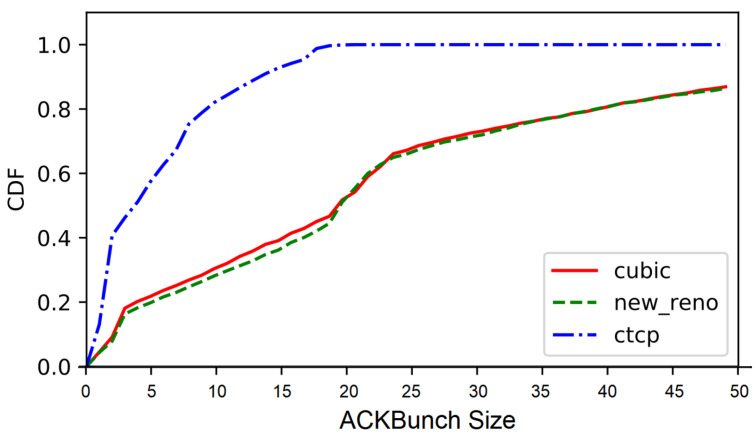
Impact of TCP congestion control algorithms.

Figure 8 depicts the ACKBunch CDF for varied TCP congestion control algorithms: TCP CUBIC, New Reno, and compound TCP [27]. Notably, compound TCP, which is used in Windows OS, shows smaller ACKBunch size, compared with the other two. Specifically, compound TCP has 30% less ACKBunch size compared with TCP CUBIC. One key observation is that compound TCP has less ACKBunch size compared with other TCP congestion control algorithms, yet the slopes or differences between CDF-2 and CDF-12 are quite similar.

Based on this observation, we propose the enhanced *Weigh-In-Motion* Algorithm for better traffic classification in Appendix A. Algorithm A1 (Appendix A) exploits the empirical distribution observed in the experiments with different TCP congestion control algorithms. Algorithm A1 shares the same procedures with Algorithm 1 except the procedure *OnReceiveTCPAck()*. Unlike Algorithm 1, *OnReceiveTCPAck()* uses the difference between CDF-2 and CDF-12 as illustrated in lines 7 to 13 in Algorithm A1. This procedure is shown to effectively filter out the exceptional traffic pattern of the *compound TCP* congestion control algorithm. We further present the effectiveness of Algorithm A1 in Appendix A.
**Algorithm 1** *Weigh-In-Motion:* Online Sequential Hypothesis Test for TCP-ACK flow *m*
**procedure** *Initialize()*  1:n,l←0  2:C←1 // for counting ACKBunch size $|\Lambda^{m}|$  3:$t_{last}\leftarrow 0$  4:$\theta_{0}\leftarrow 0.5$**procedure** *OnReceiveTCPAck()*    1://*EventRX: Upon receipt of a new TCP ACK of flow m*    2:$\tau_{t}\leftarrow {\mathrm{time}}_{current}-{\mathrm{time}}_{last}$    3:**if**$\;\tau_{t}\leq \delta =34\mu sec$ or $t_{last}==0$ **then**    4:   // the incoming TCP-ACK belongs to n-th ACKBunch    5:   // increase the ACKBunch size by one    6:   C←C+1    7:**else**    8:   //*Separation of ACKBunch*    9:   //*the TCP-ACK belongs to (n+1)-th ACKBunch*  10:  //*Processing n-th ACKBunch*  11:   n←n+1  12:   **if** C≤k(=12) **then**  13:     //*Counting # of ACKBunch whose size is less than k*  14:     l←l+1  15:   **end if**  16:   // Test whether to reject $H_{0}$ or not  17:   **TestSPRT(*n*, *l*)**  18:   // initialize the size  19:   C←1  20:   **if** l>Limit **then**  21:     //*periodically initialize the test*  22:     Initialize()  23:   **end if**  24:**end if**  25:${\mathrm{time}}_{last}\leftarrow {\mathrm{time}}_{current}$**procedure** *TestSPRT(n, l)*  1:$\hat{p}\leftarrow l/n$  2:**if**$\;n<\frac{l(\log\left(\hat{p}/\left(1-\hat{p}\right)\right)+log\left(\left(1-\theta_{0}\right)/\theta_{0}\right))-\log K}{\log\left(1-\theta_{0}\right)-\log\left(1-\hat{p}\right)}$**then**  3:   reject $H_{0}$  4:   conclude $H_{a}$:*flow u is Gbps Wi-Fi traffic*  5:**else**  6:   undetermined; do not reject $H_{0}$  7:**end if**

## 6. Performance Evaluation

In this section, we test our algorithm through real experiments, which allows us to evaluate the packet classification performance in a variety of network conditions with several different network configurations.

### 6.1. Experiment Setup

We conducted experiments with the same setup presented in Section 4 and Figure 4. For the Wi-Fi link, we used three different IEEE 802.11 APs. For IEEE 802.11g, we use ipTIME G100 [28] that does not support MIMO (Multiple Input Multiple Output). For IEEE 802.11n, we use TP-Link N750 [29] that supports MIMO 3×3. Finally, for IEEE 802.11ac, we use two types of APs: TP-Link1750 AC1750(C7) that supports MIMO 3×3 and TP-Link C3150 that supports MIMO 4×4 [29].

Both hosts using Ethernet and Wi-Fi use the MacOS operating system. We varied six network parameters for Wi-Fi: Chipset vendor (TP-Link AC1750 and TP-Link C3150), AP vendor (Broadcom and Realtek), TCP congestion control (TCP Cubic, New Reno, and Compound TCP [27]), TCP delayed ACK option (0 or 2), and memory size (default, and 2×). Meanwhile, the Ethernet link utilizes the CUBIC TCP congestion control, and ACK delay option 2, with default memory size. For each parameter combination, we have measured out-bounding traffics at p2 in Figure 4 and constructed test data sets, consisting of 750 Wi-Fi data sets, and 250 Ethernet data sets, a total of 1000 data sets. We have used two different traffic types in the experiments: FTP and HTTP. In our experiments, we used HTTP to download large files from a local Web server.

The worst-case scenario is when the Ethernet link is performing poorly while the Wi-Fi link condition is healthy. Here, the two links should show similar link speeds, thus difficult to distinguish between the two links through traffic classification. Therefore, we render cross-traffic at the Ethernet link so that the link is under saturated traffic.

### 6.2. Evaluation Results

It is important for our proposed approach to be not limited to a specific environment, but operate with high accuracy in a real world where there are various environmental variables including target devices’ operating systems, network parameters/options, Wi-Fi chipset vendors, and so on. To verify how ACKBunch is affected by environmental variables, we conduct various experiments to characterize and analyze the properties of ACKBunch.

**Figure 9:**
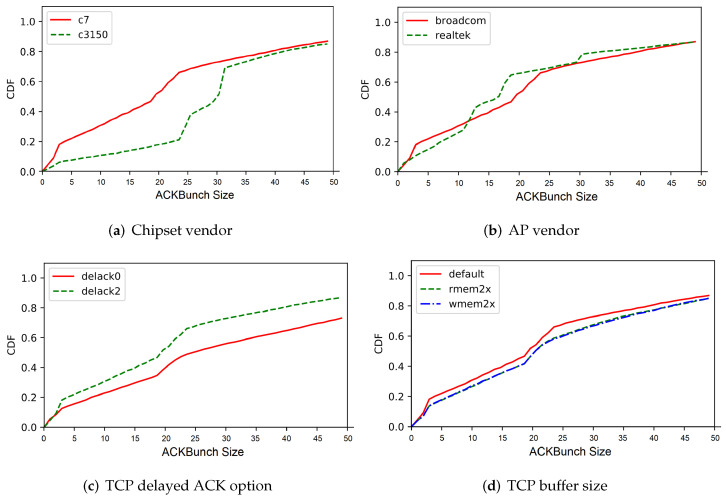
CDF of (**a**) Inter-packet arrival time (IAT), (**b**) Inter-ACK time, (**c**) round-trip-time (RTT), and (**d**) TCP buffer size.

Figure 9 plots the CDF of ACKBunch size, with varied network parameters. Figure 9a presents the ACKBunch size CDF for two IEEE 802.11ac chipsets: TP-Link1750 AC1750(C7) and TP-Link C3150 [29]. C3150 shows larger ACKBunch size since it uses MIMO 4×4, while C7 uses MIMO 3×3, since the throughput will typically increase with more MIMO antennas.

Figure 9b shows that the ACKBunch size are generally similar for each AP vendor (Broadcom and Realtek). In result, we observe that the AP vendor does not have much effect on the ACKBunch size.

If the TCP delayed ACK option is turned on, the TCP receiver will wait for a certain amount of time (e.g., 500 ms), before sending an ACK. If another data packet arrives, or the timer expires, then the receiver will immediately transmit the ACK. This option reduces the number of ACKs, as well as increasing the inter-ACK delay. Therefore, the TCP delayed ACK option tends to give less ACKBunch size, as we witness in Figure 9c. Nevertheless, we can observe that our classification criterion presented in Section 5 is valid for both options.

In addition, we have investigated the effect of TCP buffer size (receive, send) on the distribution of ACKBunch size. Figure 9d shows that the increased TCP buffer size will give more opportunities for ACKBunch, thus larger ACKBunch size. However, its difference in value was not significant, so it did not affect the classification performance.

**Figure 10:**
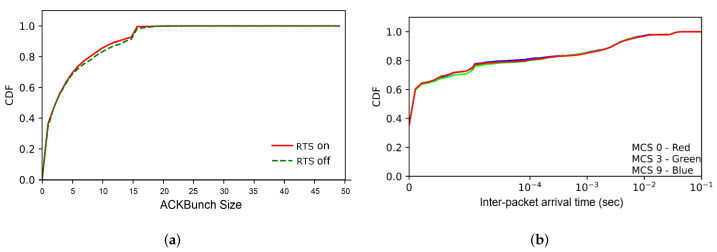
Effect of IEEE 802.11 conditions: (**a**) RTS/CTS option, and (**b**) MCS values.

We have tested the effect of RTS/CTS (Request-to-Send/Clear-to-Send) option (on/off) and MCS (Modulation and Coding Scheme) of IEEE 802.11. Figure 10a shows the ACKBunch size CDF with different RTS/CTS options, and Figure 10b shows the distribution of inter-packet (ACK packets) arrival time measured at the monitoring point. The results show that these factors do not significantly affect the proposed metric, ACKBunch.

Table 2 evaluates the accuracy of *Weigh-In-Motion* described in Algorithm 1 with 1000 data sets. The decision parameter in Equation (5), *K*, is varied by $10^{4},10^{5}$, and $10^{6}$. The results show the significant impact of *K* on the detection accuracy and detection time. When *K* = $10^{5}$, the correct identification ratio is at least 96%. However, when *K* is relatively small ($10^{4}$), the accuracy drops significantly. In a more conservative environment *K* = $10^{6}$, the average identification accuracy was 97.8%, which is not a significant increase. In other words, at least 2–3% of inaccuracies existed in this experimental environment. In Section 5.2, we discussed the main cause of this inaccuracy and proposed an enhanced algorithm to overcome this problem.

Table 3 shows the identification results with three different design parameters of *K*. As shown in Table 2 and Table 3, a higher value of *K* improves the detection accuracy, but incurs higher delay in identification. Algorithm A1 achieves very high accuracy (more than 99 percent) very quickly (average of 0.7278) with $K=10^{5}$. Although Algorithm 1 shows smaller average identification time compared to Algorithm A1, when *K* is relatively small ($10^{4}$), the accuracy drops significantly. In a conservative configuration with $K=10^{6}$, the identification accuracy is 100 percent, but the delay increases significantly.

**Figure 11:**
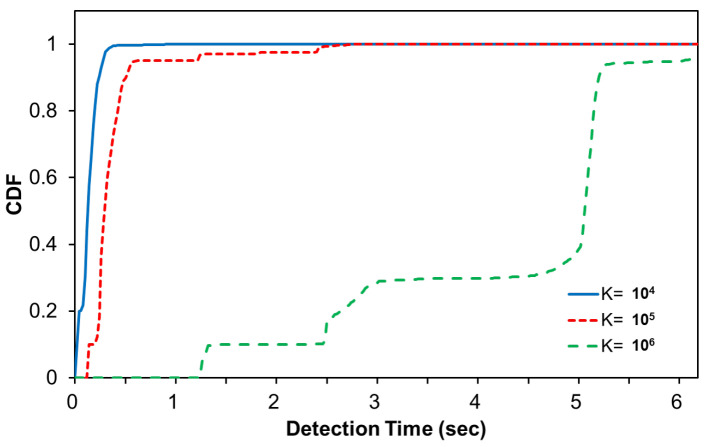
Impact of *K* on detection time.

Figure 11 compares the identification time for different design parameter *K*. There is a trade-off in selecting *K*; a larger *K* increases the detection accuracy, but requires a longer detection time.

## 7. Discussions

This section provides relevant discussion points for our approach.

**Design philosophy and limitation.** Our approach relies on TCP ACK information, therefore does not accommodate traffic streams other than TCP. The design philosophy and the envisioned use of our approach are not to totally replace all classification solutions deployed in existing IDS systems, but to use our proposed method as an add-on component to operate as an efficient classifier for TCP-based traffic.

**802.11ax and multi-user scenarios.** As discussed in Section 2.2, the state-of-the-art Wi-Fi standard 802.11ax, also known as Wi-Fi 6, introduces several major breakthroughs [17], including downlink/uplink OFDMA, UL MU-MIMO, higher-order modulation, and enhanced spatial re-use. However, our current focus in this work is on the A-MPDU functionality used generally in 802.11 standards.

**Data-driven approach.** As we introduced in Section 2.1, research on traffic classification using machine learning and deep learning have obtained growing attention. As future work, we also consider a data-driven approach such as machine learning and deep learning to tackle the same problem of this paper. As a preliminary experiment, we processed a training dataset consisting of 1000 measurement traffic sets and performed supervised learning using linear regression algorithms and Support Vector Machine (SVM). We obtained meaningful results with the hit rate accuracy of 99.9% although 1000 data sets are not sufficient for machine learning.

**EU Regulation.** One thing to note is that, if our method is applied in the middlebox to block some type of traffic (e.g., 802.11ax), this approach would be in violation of the EU Regulation [30] in combination with the provision of an Internet access service. We would like to acknowledge the editor for this comment.

## 8. Conclusions

In this paper, we presented *Weigh-In-Motion*, a lightweight online classification scheme that analyzes the traffic patterns observed at the middleboxes and infers whether the traffic originated from high-speed Wi-Fi devices. Our proposed approach is based on a new simple and practical metric *ACKBunch* that captures the unique characteristics of high-speed Wi-Fi, in particular A-MPDU frame aggregation. The effectiveness of the proposed scheme is evaluated via extensive real experiments, demonstrating its capability of accurately identifying wireless traffic from/to Gigabit 802.11 devices.

We plan to extend our work to analyze and characterize such the 802.11ax key enhancements. We are currently working to understand the unique characteristics of 802.11ax traffic in the presence of multiple users with a completely different approach from the current work.

We would also like to extend our work to the data-driven approach, as discussed in Section 7.

## Figures and Tables

**Table 1:** Slot time and DIFS value in IEEE 802.11.

Standard	Slot Time (μs)	DIFS (μs)
802.11b	20	50
802.11a	9	34
802.11g	9 or 20	28 or 50
802.11n (2.4 GHz)	9 or 20	28 or 50
802.11n (5 GHz)	9	34
802.11ac	9	34

**Table 2:** Identification Performance of Algorithm 1 (δ=34μs).

	$K=10^{4}$	$K=10^{5}$	$K=10^{6}$
Accurate Identification Rate (%)	28.9%	96.4%	97.8%
False Positive Ratio (%)	5.4%	0.1%	0.0%
Average of Identification Time (s)	0.1034	0.6803	3.724

**Table 3:** Detection performance of Enhanced *Weigh-In-Motion* Algorithm (δ=34μs).

	$K=10^{4}$	$K=10^{5}$	$K=10^{6}$
Accurate Identification Rate (%)	83.6%	99.9%	100.0%
False Positive Ratio (%)	13.9%	0.1%	0.0%
Average of Identification Time (s)	0.1075	0.7278	3.7314

## Data Availability

Not applicable.

## Abbreviations

The following abbreviations are used in this manuscript:

A-MPDU	aggregate medium access control (MAC) protocol data unit
AP	Access Point
CDF	cumulative distribution function
DCF	Distributed Coordination Function
IAT	Inter-packet arrival time
IDS	Intrusion detection system
IFS	inter frame space
MAC	Medium access control
MPDU	MAC-level protocol data unit
PPDU	Physcial-layer protocol data unit
RTT	Round Trip Time
WLAN	Wireless LAN
$\Lambda^{\left(m\right)}$	ACK Bunch for TCP flow *m*
$|\Lambda^{\left(m\right)}|$	length of *ACKBunch* $\Lambda^{\left(m\right)}$

## Appendix A

In Section 5.2, we discussed the main cause of the inaccuracy of *Weigh-In-Motion* described in Algorithm 1. In this Appendix, we depict the enhanced *Weigh-In-Motion* Algorithm for better traffic classification. It is designed based on the observation that compound TCP has less ACKBunch size compared with other TCP congestion control algorithms, yet the slopes or differences between CDF-2 and CDF-12 are quite similar. Algorithm A1 exploits the empirical distribution observed in the experiments with different TCP congestion control algorithms.

Algorithms 1 and A1 share the same procedures except the procedure *OnReceiveTCPAck()*. In Algorithm A1, *OnReceiveTCPAck()* uses the difference between CDF-2 and CDF-12 as illustrated in lines 7 to 13 in Algorithm A1. As observed in Section 6, Algorithm A1 is shown to effectively filter out the exceptional traffic pattern of the *compound TCP* congestion control algorithm.

**Algorithm A1***Weigh-In-Motion:* Online Sequential Hypothesis Test for TCP-ACK flow *m* (Modification)
**procedure***Initialize()* 1: //invoke *Initialize()* in **Algorithm 1** 2: **Algorithm 1:***Initialize()* 3: $l_{2},l_{12}\leftarrow 0$ **procedure***OnReceiveTCPAck()* 1: //*EventRX: Upon receipt of a new TCP ACK of flow m* 2: $\tau_{t}\leftarrow {\mathrm{time}}_{current}-{\mathrm{time}}_{last}$ 3: **if** $\tau_{t}\leq \delta =34\;\mu$s or $t_{last}==0$**then** 4: C←C+1 5: **else** 6: n←n+1 7: **if** C≤k(=2) **then** 8: $l_{2}\leftarrow l_{2}+1$ 9: **even if** C≤k(=12) **then** 10 $l_{12}\leftarrow l_{12}+1$ 11: **end if** 12: // //*Modified test: use the difference between CDF-2 and CDF-12* 13: $l\leftarrow max(l_{12}-l_{2},0)$ 14: // Test whether to reject $H_{0}$ or not 15: **Algorithm 1:***TestSPRT(n, l)* 16: // initialize the size 17: C←1 18: **if** l>Limit **then** 19: //*periodically initialize the test* 20: Initialize() 21: **end if** 22: **end if** 23: ${\mathrm{time}}_{last}\leftarrow {\mathrm{time}}_{current}$
